# Action-Perception Coupling and Near Transfer: Listening to Melodies after Piano Practice Triggers Sequence-Specific Representations in the Auditory-Motor Network

**DOI:** 10.1093/cercor/bhaa018

**Published:** 2020-05-21

**Authors:** Örjan de Manzano, Karen L Kuckelkorn, Karin Ström, Fredrik Ullén

**Affiliations:** Department of Neuroscience, Karolinska Institutet, 17177, Stockholm, Sweden

**Keywords:** action-perception coupling, fMRI, music, MVPA, sequence learning

## Abstract

Understanding how perception and action are coupled in the brain has important implications for training, rehabilitation, and brain–machine interfaces. Ideomotor theory postulates that willed actions are represented through previously experienced effects and initiated by the anticipation of those effects. Previous research has accordingly found that sensory events, if previously associated with action outcomes, can induce activity in motor regions. However, it remains unclear whether the motor-related activity induced during perception of more naturalistic sequences of actions actually represents “sequence-specific” information. In the present study, nonmusicians were firstly trained to play two melodies on the piano; secondly, they performed an fMRI experiment while listening to these melodies as well as novel, untrained melodies; thirdly, multivariate pattern analysis was used to test if voxel-wise patterns of brain activity could identify trained, but not novel melodies. The results importantly show that after associative learning, a series of sensory events can trigger sequence-specific representations in both sensory and motor networks. Interestingly, also novel melodies could be classified in multiple regions, including default mode regions. A control experiment confirmed these outcomes to be training-dependent. We discuss how action-perception coupling may enable spontaneous near transfer and action simulation during action observation.

## Introduction

Understanding how perception and action are coupled in the brain has important implications for training, rehabilitation, and designing brain-machine interfaces (Flor and Diers 2009; Aflalo et al. 2015). According to ideomotor theory, which has gained influence in recent years, willed actions are learned by trial and error based on perceived outcomes; therefore, the brain is organized such that at an abstract level, perception and action are represented by a common code (Prinz 1990). This means that observed, imagined, and performed actions all exist in the same representational domain, essentially making action selection and prediction of outcomes two sides of the same coin (Herwig 2015). Empirical support for this principle comes from studies of reciprocal influences between action and perception. If sensory events occurring during an action match expectations based on previous experience, both perception and performance may be boosted; in contrast, if sensory events are incongruent with the act, interference may occur (reviewed in Novembre and Keller 2014; Herwig 2015). For example, Keller and colleagues showed in a series of experiments that musicians planned finger movement sequences faster when response keys and auditory feedback were congruent with the design of their instruments, and that this effect got stronger with increased musical expertise (Keller and Koch 2008). They also demonstrated that in musicians, the anticipation of auditory sensory events can improve timing and economy of movements (Keller et al. 2010). Using transcranial magnetic stimulation, Stephan et al. (2018) showed that when nonmusicians heard melodies previously practiced on a keyboard, motor-evoked potentials were enhanced such that the anticipation of tones cued the corresponding finger-movements. Neuroimaging has further illustrated how action-perception coupling may develop through associative learning (e.g., Haslinger et al. 2005) and found a substantial overlap between brain regions involved in observing, imagining, and executing familiar actions (Hardwick et al. 2018). For example, Lahav et al. (2007) showed that after nonmusicians practiced playing a musical piece on the piano for 5 days, listening to the trained musical piece evoked additional brain activity, compared with novel material, in the inferior frontal gyrus (IFG) and premotor cortex, which was interpreted as training-dependent activation of motor representations. Herholz et al. (2016) further demonstrated, after a 6-week piano intervention, an increase in brain activity in the dorsal premotor area (PMD) and parietal cortex during both listening and auditory imagery of trained versus untrained melodies.

However, it remains unclear whether the motor-related activity induced during passive perception of action sequences actually represents “sequence-specific” information at a similar level of complexity. Wiestler and Diedrichsen (2013) have shown that performed movement sequences can be classified according to voxel-wise patterns of brain activity in several motor areas. This observation, together with the basic tenets of ideomotor theory, suggests that perception of learned action sequences should trigger sequence-level motor representations. Apsvalka et al. (2018) found support for this prediction using visually observed sequences. Still, it is not clear how such action-perception depends on training and if it extends to the auditory-motor domain. Here, these issues are addressed using a group of nonmusicians who were firstly trained to play two simple melodies differing only in ordinal structure on the piano; secondly, an fMRI experiment was carried out where they listened to these melodies as well as novel (untrained) melodies; and thirdly, multivariate pattern analysis (MVPA) was used to test if patterns of brain activity could be used to distinguish between the trained, but not the novel melodies. The PMD was chosen as region-of-interest (ROI) since this region has consistently been linked to the perception, learning, production, and creative generation of ordinal/melodic sequences (Schubotz and von Cramon 2001; Bengtsson et al. 2004; Bischoff-Grethe et al. 2004; Bengtsson and Ullén 2006; de Manzano and Ullén 2012). The posterior superior temporal gyrus (pSTG) was chosen as a secondary ROI to replicate that melodic gestalt is represented in the auditory cortex (Schindler et al. 2013). Lastly, a moving-ROI/searchlight analysis was performed to explore auditory-motor coupling in additional brain regions.

## Materials and Methods

The study comprises two experiments that were carried out in a consecutive fashion as the second experiment served to follow up on an unexpected finding in the first experiment (Interim discussion). The respective participant samples are henceforth referred to as the experimental group (Experiment 1) and the control group (Experiment 2).

## Experiment 1

### Participants

In total, 15 individuals were recruited to the study via open advertisements. A corresponding sample size has been found sufficient in similar studies using MVPA (Schindler et al. 2013; Wiestler and Diedrichsen 2013; Apsvalka et al. 2018; Yokoi et al. 2018), which leverages primarily on collecting a substantial amount of data within person. The inclusion criteria were that participants should be adult (>18 years old), be right handed, have less than two years of musical training in childhood, have no musical training as adults, have no musical training on the piano, have no history of neurological or psychological disease, and be able to participate in MRI in accordance with general safety regulations. Three participants were excluded from the study post MRI; one participant was scanned as a pilot to test the experimental procedure and evaluate efficacy of the design for MVPA and was discarded due to changes in participant instructions and optimizations of the experimental procedure; data from one participant were corrupted due to a technical failure of the MRI scanner, and one participant was excluded after the routine neurological screening, which was performed by a neuroradiologist. Hence, data from 12 participants (age *M* = 28 ± 5.5 years; 4 female) were included in the final analyses. The experimental procedures were undertaken with written informed consent of each participant, conformed to The Code of the World Medical Association (Declaration of Helsinki) and ethically approved by the Regional Ethical Review Board (Dnr: 2017/2304-32). Participants were reimbursed with 1000 SEK.

### Auditory Stimuli

**Figure 1:**
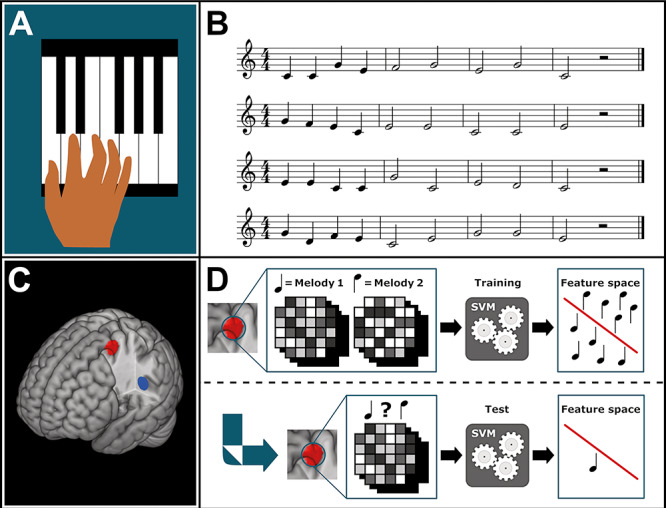
Methods illustrations. Panel *A*: The relevant piano keys and fingering. Panel *B*: The four melodies. Panel *C*: The two ROIs (red—PMD; blue—pSTG). Panel *D*: Illustration of the MVPA classification process. The upper section illustrates how patterns of brain activity from a ROI is given to the classifier as training material (SVM—support vector machine), in order to derive a decision boundary between the two melodies in multivariate feature space. The lower section illustrates how additional patterns are used to test the accuracy of the classifier after it has been trained.

Four original melodies were composed for the experiment using the software Logic Pro X and the included Bösendorfer Grand Piano sound samples (by one of the authors, KS). All melodies were composed to be played with one hand and fixed fingering and were thus based on the 5 first notes of the C major scale (C4, D4, E4, F4, G4), and had the same meter (4/4 time), rhythm (4 quarter notes followed by 5 half notes and a half rest), and tempo (115 bpm) (see Fig. 1, panels *A* and *B*). The final rest had a duration of 350 ms, giving each melody a total duration of 8 s. To ensure a sufficient melodic dissimilarity between melodies, any three-note combination only appeared once across all melodies. Furthermore, no particular note transition (e.g., C4 → D4) appeared at a given position in more than one melody.

### Piano Training

The participants of the experimental group were invited to a piano training session one day before their respective MRI experiment. During this training session, each of the participants learned to play two (out of the four) melodies on a MIDI keyboard. The keyboard was connected to a laptop with the Logic Pro X (Apple, Inc.) recording studio software installed and ready to deliver Bösendorfer Grand Piano sound samples through the laptop speakers. The assignment of melodies as trained and novel was counterbalanced across participants. The training sessions would begin by letting the participant listen to one of the selected melodies played from the laptop, after which an instructor would demonstrate how to play the corresponding melody on the keyboard. The participant was then told to place the fingers of the right hand on the correct keys (C4-G4) and try to mimic the instructor, who would play the melody repeatedly at a slow tempo an octave lower on the keyboard. When the participant had learned to play the melodic sequence independently and reliably, the participant was asked to play the melody for 20 min (about 145 repetitions) in tempo with a metronome (115 bpm). This procedure was subsequently repeated for the second melody. After the two melodies had been practiced, the participant was asked to play the two melodies in an alternating fashion for another 20 min. As a final test, the participant was asked to play each melody three consecutive times correctly. All participants passed this test. The training dose was assumed to be sufficient based on TMS studies showing robust training-specific action-perception coupling after piano-keyboard practice as brief as 30—40 min (D'Ausilio et al. 2006; Stephan et al. 2018).

## Data Collection/MRI Experiment

### Prescan Preparations

All MRI sessions were performed at the MR Research Center of the Karolinska Hospital. Upon arrival, the participants first underwent a second training session. They were placed in a quiet room where they practiced their assigned melodies in tempo with a metronome (115 bpm) for 30 min, using the same instrumental setup as in the first session.

### Experimental Procedure

Participants were scanned in a supine position. Earplugs and headphones were used in combination to reduce the noise of the scanner, while allowing auditory feedback and communication with the experimenters in the control room between sessions. The fMRI procedure included the main listening paradigm and a finger tapping paradigm for the localization of functional ROIs. Detailed instructions of each paradigm (as described below) were given to the participants before scanning.

The listening experiment was carried out in 10 sessions, each composed of 32 trials. A trial would consist of the presentation of one melody (8 s) followed by two seconds of silence. The auditory stimuli were identical to those composed for the first practice session (see Auditory stimuli). Four trials with the four different melodies formed one set of trials. The 32 trials of a session thus consisted of eight such sets of four trials each (320 s in total). To minimize expectancies on transitions between melodies, the trial order was permuted such that the same melody was never played two times in a row, and the distribution of transitions between different melodies during a session was made to be as uniform as possible. Five permutations of set orders were created, each used twice across the 10 sessions (53 min 20 s in total). The short interval between melodies and the brief rest was an intentional tradeoff that compromised the efficacy of the design for traditional voxel-wise univariate testing (e.g., contrasting average brain activity between trained and novel melodies as the BOLD response induced by one melody overlaps initially with the next melody), in favor of collecting more samples for machine learning and classification (see Data analysis). A preliminary analysis of the obtained pilot data suggested that the design would nonetheless be effective for MVPA analyses (i.e., melodies could be classified despite the presumed overlapping BOLD-responses). The instructions given to participants were to pay attention to the currently played melody and to evaluate whether it was trained or novel. This task was simply devised to keep participants focused on the melodic structure of the presented stimuli throughout the experiment. No behavioral response was obtained in order not to confound motor representations evoked by passive listening. All participants were additionally instructed to keep their eyes open and fixate on a black crosshair presented centered on a white background (back-projected on a screen behind the scanner and viewed by the participants though a periscope mirror attached to the head coil) and to remain still throughout the experiment and particularly not to move their fingers. An adhesive tape was also placed around the fingers of the right hand. The participants were further instructed not to try to figure out how to play the novel melodies while these were presented.

The finger tapping paradigm was carried out in a single session after the listening experiment. The session was composed of 30 s rest, 2 min of finger tapping, and another 30 s of rest (3 min in total). Before this session, the adhesive tape was removed from the right hand. Visually presented words (black font, centered on a white background) would indicate the conditions (Rest/Move). During the Rest condition, the participants were to remain still, keep their eyes open and fixate on the presented word. During the Move condition, participants were also to keep their eyes open and fixate on the word, but also to tap the fingers of their right hand on the gurney in tempo with a metronome (115 bpm), and try to produce a “random” tapping sequence.

### MRI Scanning Parameters

Imaging was performed using a 3-T scanner (Discovery MR750w 3.0T, GE Healthcare, Chicago, Illinois, USA) with a 32-channel head coil. Functional imaging data were collected using a gradient echo, echo-planar (EPI) T2*-weighted sequence with blood oxygenation level-dependent (BOLD) contrasts. The following parameters were used: repetition time (TR) = 2.08 s; echo time (TE) = 30 ms; field of view (FOV) = 23 cm; matrix size = 76 × 76; voxel size = 3 × 3 × 3 mm^3^; slice thickness = 3 mm; slice spacing = 0.2 mm; flip angle = 80°. Whole brain image volumes were constructed from 43 contiguous axial slices in an interleaved slice order. Ten “dummy” image volumes were acquired at the beginning of each session, to allow for T1 equilibration effects, but not saved. A high-resolution, three-dimensional spoiled gradient echo T1-weighted anatomical image was acquired in axial slice orientation: TR = 5.45 ms; TE = 2.36 ms; inversion time (TI) = 450 ms; FOV = 24 cm; matrix size = 240 × 240; voxel size 1 × 1 × 1 mm^3^; flip angle = 12°. A T2-weighted 3D fast spin echo fluid-attenuated inversion recovery image was also acquired: TR = 8000 ms, TE = 115 ms, TI = 2258 ms, FOV = 27 cm; matrix size = 224 × 224, voxel size = 1.2 × 1.2 × 1.2 mm^3^, variable flip angle.

## Data Analysis

### Preprocessing of MRI Data

FMRI data were preprocessed using the SPM12 software package (Wellcome Department of Imaging Neuroscience, London, UK) implemented in MATLAB R2017b (MathWorks, Inc.). For each participant, all fMRI image volumes were slice time corrected, realigned to the first image of the first session (Friston et al. 1995) and unwarpped to remove residual variance caused by movement (Andersson et al. 2001). Thereafter, the unwarpped images and the T2-weighted image were coregistered to the T1-weighted image (Ashburner and Friston 1997). To estimate the deformation field for the normalization of functional images and the anatomical images, the T1-weighted and the T2-weighted images were jointly segmented (Ashburner and Friston 2005). The normalized images were subsequently smoothed using a Gaussian kernel with a full-width-at-half-maximum (FWHM) of 8 mm.

### Univariate Analyses of fMRI Data

In order to calculate the beta estimates that were used as input in the MVPAs, a first/subject-level univariate analysis of the fMRI data from the listening paradigm was performed. The fMRI data were modeled using a general linear model (GLM) and the standard hemodynamic response function. The first-level GLM included four regressors of interest for each session, representing the onsets and durations of the four melodies. The high-pass filter was set to 140 s (i.e., the Nyquist rate, or 2 × the maximum period between an experimental condition and its repeat). This analysis resulted in one beta image per melody per session, which was later used as input in the MVPAs.

In the analysis of the finger tapping paradigm, normalized and smoothed fMRI data were used. The first-level GLM analysis modeled the single Move condition and used the default high-pass filter of 128 s. A second-level (group) analysis was performed on the contrast between Move and implicit baseline (including Rest) using a one sample *t*-test and an uncorrected statistical threshold of *P* < 0.001.

### Definition of Functional ROIs and Gray Matter Masks

The functional ROIs and MNI space gray matter mask were recalculated after Experiment 2 with data from all participants not to bias the analyses towards a specific sample. All results presented in this article are based on those final masks. To locate the coordinates of peak activity for the left PMD in the statistical parametric map resulting from the second-level analysis of the finger tapping data, preliminary coordinates were first obtained from the meta-analysis by Mayka et al. (2006). The nearest local maxima from each of these preliminary coordinates were assumed to be the sample-specific coordinates for the left PMD (*x* = −30, *y* = −6, *z* = 64) in the present dataset. With regard to the left pSTG, the ROI center coordinate was defined as the local maximum within the pSTG with highest peak activity (*x* = −42, *y* = −34, *z* = 18). This matches well with where Schindler et al. (2013) found neural representations of melodic gestalt. While MVPA is increasingly becoming more mainstream, there is still no unanimous opinion on how to specify some variables for the analysis. This includes ROI size, particularly when the ROI is not identified as a specific anatomical region but as in the present case, as an area of certain functional significance. A number of factors such as spatial smoothing and individual differences in for instance task engagement and BOLD response play a role, which makes it difficult to determine optimal ROI size a priori. Furthermore, it has been found and replicated that classification accuracy tends to increase with the number of voxels involved in an ROI and then saturate for larger ROI sizes (Ku et al. 2008; Misaki et al. 2010). Intuitively, classification should improve as long as informative voxels are added to the analysis. Consequently, it can be recommended to assess how sensitive the outcomes are to the number of voxels that are included when using fixed ROIs. A searchlight analysis is less affected by this since the moving ROI will ultimately have included all informative voxels in the analysis mask (although not necessarily all at once). Thus, three spherical binary ROI masks with radii of 4, 6, and 8 mm, containing 33, 123, and 256 voxels respectively, were created at each of the peak coordinates using the MarsBaR toolbox for SPM12 (Brett et al. 2002). That is, the analyses would be repeated with different ROI radii to assess the influence of ROI size on classification accuracy. The ROIs did not exceed the spatial distribution of the functional outcomes of the finger tapping paradigm. The left hemisphere 8 mm ROIs are illustrated in Figure 1 (panel *C*). ROIs for the right PMD and right pSTG were created by flipping the left hemisphere ROIs to the right hemisphere. Next, the group level ROIs were transformed to the native space of each participant by using the inverse of the deformation field obtained from segmentation (see Preprocessing of MRI data). A conjunction of each native space ROI and gray matter mask produced the final ROI masks. The native space gray matter mask for each participant was created by thresholding the segmented gray matter tissue probability image at 0.5, smoothing the image by 6 mm FWHM, thresholding this image at 0.2, reslicing it to match the fMRI data using the mask image created by SPM12 during the first-level analysis as a target and then performing a conjunction of the resliced image and the just mentioned target image. The above parameters were found optimal for producing a fairly inclusive gray matter mask that nonetheless excluded spurious/irrelevant voxels. An MNI space gray matter mask was created by transforming the native space gray matter masks to MNI space and then performing a conjunction of all transformed images.

### ROI-Based MVPA

All MVPAs were performed using the CoSMoMVPA toolbox (Oosterhof et al. 2016). The main purpose of the MVPAs was to test whether different trained melodies, as opposed to different novel melodies, could be differentiated from one another using a machine learning-based classifier, trained and tested on data from the PMD. In addition, it was expected that both trained and novel melodies could be identified based on the classification of activity in the pSTG. The MVPAs were based on the beta images obtained from the first-level analysis of the listening paradigm and performed for each category of samples (trained and novel) and each ROI. The data were first demeaned to ensure that classification would not simply be driven by mean differences in activity between samples (i.e., melodies). A linear support vector machine (Chang and Lin 2011) was then used in conjunction with a leave-two-out cross-validation scheme for classification, i.e., eight and two sessions were selected for training and testing, respectively, in each iteration of the validation process. The method is illustrated in Figure 1 (panel *D*). The mean accuracy of classification across iterations was used as outcome. The within-participant chance-level performance of classification was estimated using first-level permutation testing and 10 000 permutations. The mean of the permutation distribution was used to define the chance level. Second-level analyses were performed using one-tailed one-sample *t*-tests (since only above-chance outcomes could be expected), based on within-subject paired differences between true scores and chance scores. FDR-correction (0.05) for multiple comparisons (Benjamini and Hochberg 1995) was performed based on the number of ROIs, i.e., 2 categories (trained and novel melodies) × 4 brain regions (bilateral PMD and pSTG) × 3 ROI sizes (4, 6, 8 mm). Given the dependency and spatial overlap between the ROIs, correcting for all comparisons could be regarded as conservative. The effect of ROI size on outcomes was evaluated with a repeated measures mixed model (subject and ROI as random factors) implemented in R (package lmertest) and a two-tailed statistical threshold of *α* = 0.05.

### Searchlight MVPA

In addition to the ROI-based MVPAs, searchlight-based MVPAs were performed to explore representations of melodic structure outside the predefined ROIs. The same classifier and cross-validation scheme was used as in the ROI-based MVPAs, together with a 4 mm searchlight. This searchlight size has been shown to give consistent and good performance (Kriegeskorte et al. 2006). The search space was limited to the native space gray matter masks. The resulting whole-brain classification accuracy maps were subsequently transformed to MNI-space. Within-group second-level analyses were performed using one-sample *t*-tests (expecting above chance outcomes). The results were corrected for multiple comparisons using Threshold-Free Cluster Enhancement (TFCE; Smith and Nichols 2009) through Monte Carlo simulations (Oosterhof et al. 2016) and a one-tailed statistical threshold of *α* = 0.05.

## Results

### ROI-Based MVPA

Confirming our hypothesis, the trained melodies could be classified with above chance accuracy based on activity in the PMD and pSTG (see Table 1). Contrary to our expectations, however, also the novel melodies could be classified with above chance accuracy in the left PMD and to some extent in the pSTG. As can be deduced from Table 1, there was an influence of ROI size on classification accuracy, though it was not found to be systematic in the repeated measures analysis.

**Table 1:** ROI-based classification results in Experiment 1 based on trained melodies and on novel melodies, derived from difference scores of classification accuracy (true scores − chance scores)

		LPMD			RPMD			LpSTG			RpSTG		
	*r* (mm)	Δ (%)	*P*	*d*	Δ (%)	*P*	*d*	Δ (%)	*P*	*d*	Δ (%)	*P*	*d*
Trained vs.	4	**7.2**	**0.026**	**0.86**	**7.0**	**0.041**	**0.65**	**4.7**	**0.041**	**0.64**	**5.0**	**0.030**	**0.79**
Trained	6	**10.0**	**0.007**	**1.38**	**7.9**	**0.041**	**0.66**	2.8	0.127	0.40	**3.8**	**0.047**	**0.61**
	8	**8.7**	**0.026**	**0.83**	**8.6**	**0.038**	**0.69**	**4.8**	**0.026**	**0.88**	**4.6**	**0.035**	**0.72**
Novel vs.	4	**7.2**	**0.011**	**1.17**	3.3	0.127	0.39	0.3	0.437	0.05	1.2	0.185	0.31
Novel	6	**7.0**	**0.026**	**0.83**	1.1	0.308	0.16	4.1	0.066	0.53	1.6	0.209	0.26
	8	**5.1**	**0.032**	**0.75**	1.6	0.185	0.30	3.8	0.051	0.58	**3.5**	**0.026**	**0.94**

The table displays FDR-corrected *P*-values.

*r* = ROI radius, Δ = difference score (true **−** chance accuracy), *d* = effect size (Cohen’s *d*), LPMD = left dorsal premotor area, RPMD = right dorsal premotor area, LpSTG = left posterior superior temporal gyrus, RpSTG = right posterior superior temporal gyrus

**Figure 2:**
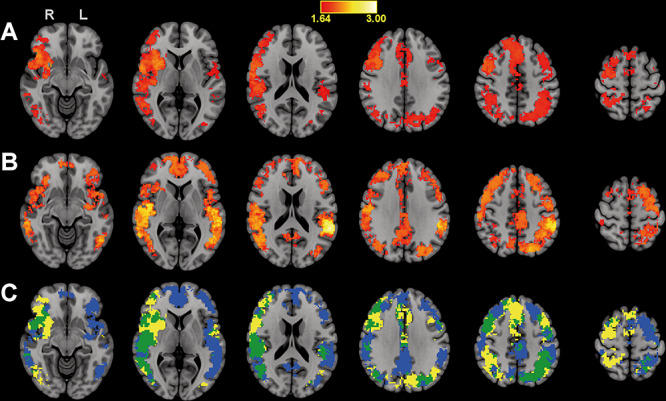
Searchlight classification results based on trained melodies and on novel melodies. Panel *A*: Significant classification accuracy (*z*-scores) of trained melodies. Panel *B*: Significant classification accuracy (*z*-scores) of novel melodies. Panel *C*: In yellow, areas where trained melodies could be classified above chance level; in blue, areas where novel melodies could be classified above chance level; in green, areas where both trained and novel melodies could be classified above chance level. R = right hemisphere, L = left hemisphere.

### Searchlight MVPA

The results of the searchlight MVPAs are illustrated in Figure 2. These results extend the ROI-based analyses by showing that both trained and novel melodies (Fig. 2, panels *A* and *B*, respectively) could be classified with above chance level accuracy within an extensive set of brain regions involved in the perception and performance of melodic content. The largest overlap of significant searchlights, i.e., where the sequence structure of both trained and novel melodies was represented, was found in the right hemisphere, in areas corresponding to the lateral and inferior frontal cortices, dorsal and ventral premotor cortices (PMD and PMV), STG, temporal-parietal and temporal-occipital junctions, and angular gyrus (see Fig. 2, panel *C*). Additional overlap was found in the middle cingulate cortex (MCC) bilaterally, the left premotor cortex, left pSTG, and a larger cluster extending across the left parietal lobules. Unique representations of trained melodic sequences was found more broadly in the right hemisphere, in areas including the lateral and inferior frontal gyri, insula, premotor cortex, inferior and superior parietal lobules, inferior temporal-occipital junction (including a small portion of the posterior fusiform gyrus), and subcortically in the putamen. There were also unique bilateral representations in the medial frontal gyrus, supplementary/presupplementary motor areas, and MCC, as well as in the left hemisphere, mostly on the border between PMD/PMV, in the inferior parietal lobule and cuneus. Classification accuracy was highest in searchlights centered in the right putamen and in an area including the right IFG/anterior insula. In contrast, novel melodies were represented more bilaterally, with a larger extent of unique representations in areas of the left hemisphere, including the prefrontal and premotor cortices, insula, putamen, STG, the temporal-parietal and temporal-occipital junctions, and the superior parietal lobule. In addition, novel sequences were uniquely represented in midline regions of the default mode network, including the medial prefrontal cortex, posterior cingulate cortex, and precuneus. The highest classification accuracy was found in the left superior temporal and supramarginal gyri.

### Overall Differences in the Representation of Trained and Novel Melodies

Finally, we explored an additional question which was immediately suggested by the previous findings, i.e., whether the two melodic categories (trained and novel) were indeed processed as separate concepts in the experimental group. To provide an answer, additional ROI-based and searchlight MVPAs were carried out in the experimental group, to understand whether trained and novel melodies could be identified as such using similar methods as described previously, i.e., by relabeling the four melodies according to these two categories. The results from the additional ROI-based analysis can be found in Supplementary Table S1 in the Supplementary Material, which shows that the two melodic categories could be identified in the PMD and in the left pSTG, while classification in the right pSTG did not quite reach significance after correction for multiple comparisons. The additional searchlight analysis demonstrated that information distinguishing the two stimulus categories was vastly distributed and found across the superior and middle temporal cortices, the parietal cortex, the superior, lateral, and inferior frontal cortices, and cerebellum (see Supplementary Fig. S1 in the Supplementary Material).

#### Interim Discussion

The surprising ability of the classifier to differentiate between novel melodies in Experiment 1 suggested two possibilities. A first explanation could be that this finding reflected transfer effects of training. That is, practicing one set of melodies may have resulted in an improved capacity to represent melodic structures that generalize also to similar but untrained melodies. A second explanation could be that there is a training-independent common neural substrate in auditory and premotor areas for perception and performance of ordinal structures. To distinguish between these explanations, a second experiment was performed where it was tested whether a classifier could differentiate between novel melodies also in a control group, i.e., in a group of participants who had not previously learned to associate tones with specific movements (see Experiment 2). Clearly, this would be predicted under the second, but not the first explanation.

## Experiment 2

Experiment 2 was performed to test whether novel melodies could be identified in (pre-)motor regions in a control group with similar demographics but who had not previously learned to associate tones with specific finger movements (see Interim discussion).

### Participants

In total, 13 individuals were recruited to the study via open advertisements. The inclusion criteria were identical to Experiment 1. One participant was excluded from the study post MRI; this participant was scanned as a pilot to test the experimental procedure and evaluate efficacy of the design for MVPA and was discarded due to changes in participant instructions and optimizations of the experimental procedure. Hence, data from 12 participants (age M = 25 ± 3.7 years; 7 female) were included in the final analyses. The experimental procedures were undertaken with written informed consent of each participant, conformed to The Code of the World Medical Association (Declaration of Helsinki) and ethically approved by the Regional Ethical Review Board (Dnr: 2017/2304–32). Participants were reimbursed with 700 SEK.

#### Auditory Stimuli

The same four melodies were used in Experiment 2 as in Experiment 1.

## Data Collection/MRI Experiment

### Prescan Preparations

As in Experiment 1, each of the participants of the control group was assigned two melodies. Before scanning, the participants were seated in a quiet room and equipped with headphones. First, each of the assigned melodies was played 10 times. Second, the participants were tested on whether they could correctly identify the melodies (as the first or second presented) when played in a random order. All participants performed perfectly at this task. Lastly, each melody was presented again three times. The purpose of this procedure was to enable the control group to perform a similar decision task in the scanner as the previous experimental group. As in the experimental group, the assignment of melodies was counterbalanced across participants.

### Experimental Procedure

Experiment 2 was carried out in a similar fashion to Experiment 1, apart from a slight adjustment to the instructions in the listening paradigm. Instead of being asked to pay attention to the currently played melody and to evaluate whether it was a trained melody or a novel melody, the control participants were asked to evaluate whether the currently played melody was a “prelistened” melody or a novel melody. Again, this task was simply devised to keep participants focused on the melodic structure of the presented stimuli throughout the experiment. Presumably, given the extent of exposure throughout scanning, prelistened and novel melodies would be equally familiar by the end of the experiment and not differ in other respects.

### MRI Scanning Parameters

The same scanning protocols as in Experiment 1 were used in Experiment 2.

## Data Analysis

The analyses in Experiment 2 were analog to those of Experiment 1, using the terms “prelistened” and “novel” melodies, instead of “trained” and “novel” melodies. As indicated above, it was not expected that information processing would differ between the two melodic categories in the control group, but the two categories were still analyzed separately to match the data analysis of Experiment 1.

## Results

### ROI-Based MVPA

Neither the prelistened nor the novel melodies could be classified above chance level in any ROI (see Table 2). Furthermore, the two melodic categories (prelistened and novel) could not be identified with significant classification accuracy in any of the ROIs (see Supplementary Table S2 in the Supplementary Material). It can be noted that with statistical thresholds uncorrected for multiple comparisons, the categories could be identified in both the left and right pSTG using 8 mm ROIs.

**Table 2:** ROI-based classification results in Experiment 2, derived from difference scores of classification accuracy (true scores − chance scores)

		LPMD			RPMD			LpSTG			RpSTG		
	*r* (mm)	Δ (%)	*P*	*d*	Δ (%)	*P*	*d*	Δ (%)	*P*	*d*	Δ (%)	*P*	*d*
Prelistened vs.	4	−0.1	0.499	−0.01	−0.4	0.490	−0.07	0.5	0.474	0.14	0.8	0.474	0.11
Prelistened	6	−0.7	0.474	−0.13	−0.6	0.474	−0.12	1.7	0.447	0.32	1.0	0.456	0.20
	8	−1.8	0.447	−0.37	−1.7	0.456	−0.25	−0.3	0.474	−0.12	0.7	0.456	0.23
Novel vs.	4	−0.4	0.490	−0.07	3.4	0.447	0.32	2.9	0.447	0.37	7.7	0.111	0.91
Novel	6	−1.8	0.456	−0.21	0.1	0.499	0.01	0.4	0.490	0.05	4.4	0.293	0.57
	8	−1.9	0.447	−0.34	2.0	0.456	0.21	0.0	0.499	0.00	6.4	0.147	0.75

The table displays FDR-corrected *P-*values.

*r* = ROI radius, Δ = difference score (true − chance accuracy), *d* = effect size (Cohen’s *d*), LPMD = left dorsal premotor area, RPMD = right dorsal premotor area, LpSTG = left posterior superior temporal gyrus, RpSTG = right posterior superior temporal gyrus

### Searchlight MVPA

Neither the prelistened nor the novel melodies could be classified significantly above chance level in any searchlight portion of the brain. Using a “trend-level” statistical threshold of *P* = 0.1, there were informative searchlights in the right superior temporal sulcus extending into Brodmann area 42.

## Discussion

### Sequence-Level Action-Perception Coupling

In this study, MVPA was used to test if listening to melodies previously practiced on the piano induces spatial patterns of brain activity that represent sequence-specific information in the premotor cortex, specifically in the PMD. The ROI-based MVPA confirmed this hypothesis, showing that two trained melodies could be classified consistently above chance in the bilateral PMD of the experimental group. This is novel and direct evidence that associative learning of sensorimotor sequences involves *de novo* formation of action-perception coupling at the sequence level. Notably, this also means that perceptual and motor chunking, i.e., the grouping of input and output into familiar units (Miller 1956), can be intrinsically related. Anecdotally, this might explain for instance why many individuals tend to remember for instance door codes as “movement sequences” that are easily retrieved given appropriate sensory stimulation (looking at a number pad), but difficult to recall otherwise. In any case, the findings clearly demonstrate that after associative sensorimotor learning, ordinal structures of sensory information can activate sequence-specific patterns of brain activity in the motor system.

A secondary hypothesis was that both trained and novel melodies would be represented in the pSTG. As briefly mentioned in the Introduction, Schindler et al. (2013) previously found that melodic gestalt is represented in the auditory cortex. Using MVPA, the authors were able to distinguish between short melodic sequences based on patterns of brain activity in Heschl’s gyrus and the pSTG. There was consequently a clear expectation on replicating these findings in both experiments. The ROI-based MVPAs provided partial support for this hypothesis. The trained melodies could be classified in the bilateral pSTG, but accuracy was generally lower for the novel melodies and in both cases influenced by ROI radius. Individual melodies could however not be identified in the control group. There was some support for that the two general categories (prelistened and novel) could be classified, which is worth noting since the sensitivity analysis (including ROIs of different sizes) introduced variability in outcomes and inflated multiple comparisons. That is, these weaker results might indicate a form of melodic gestalt, which could potentially have been revealed with a larger sample size of the control group. Alternatively, these findings could represent the confounding effect of making and representing a decision about whether the melodies were prelistened or novel. Nevertheless, it seems that the representation of sequence structure in auditory areas, in the absence of downstream processing and feedback signals associated with action-perception coupling, was more difficult to identify. It does not appear likely however, that increasing the sample size could have helped confirm representations of melodies in the PMD in the control group. Firstly, as described in the introduction, previous studies strongly support that action-perception coupling and “resonance” in motor areas is contingent on the perceived action being part of the observer’s/listener’s motor repertoire (Rizzolatti and Craighero 2004). Secondly, the mean classification accuracies in the left and right PMD across ROIs and across participants in the control group were −0.008 and −0.009%, with effect sizes of −0.16 and −0.20 (see Table 2), which means that there was not even a hint of an effect that could be statistically amplified by adding participants. Thus, we maintain that Experiment 1 provides direct evidence for “training-dependent” formation of sequence-specific action-perception coupling.

### Transfer of Sequence-Independent Features to Novel Melodies

While the initial hypothesis was that the melodies would be perceived in a holistic fashion, and therefore only be associated with a motor representation if practiced previously on the piano, the ROI-based MVPA in the experimental group clearly demonstrated that the left PMD represented sequence-specific information also during listening to the novel melodies. This effect was not replicated in Experiment 2, where participants listened to the same melodies without previous piano practice. That is, the results from Experiment 1 could be interpreted as training-dependent implicit positive transfer of sequence-independent features that allowed for chunking of ordinal structures and auditory-motor coupling when the experimental group were presented with novel melodies (Thomas and Nelson 2001; Robertson 2007). Since this was an unexpected finding and the experiment was not designed to analyze transfer mechanisms, further studies will be required to determine whether the observed transfer resulted from similarities in acoustic or other structural features. Nevertheless, the effect was presumably facilitated by having a set of tone-finger associations that was identical in both conditions. The substantial amount of repeated exposure was likely also a contributing factor. A tentative explanation for the positive transfer, based on ideomotor theory, would be that the previously formed coupling between individual tones and finger movements caused the auditory-motor system to essentially play by ear and bind the items into sequences, even though the participants were specifically told not to imagine playing the novel melodies and reportedly did not do so. Thus, what was arguably generalized was the implicit ability to bind tones and their corresponding finger movements into chunks—a skill developed during piano practice.

### Distributed Representations of Sequence-Specific Information

The searchlight MVPA of trained melodies provided further support for training-dependent sequence-specific action-perception coupling. Informative voxels were revealed in searchlights across many regions of the large-scale auditory-motor network active during melodic/spatial pitch processing and piano playing (Bengtsson and Ullén 2006). This wide distribution of informative activity patterns was beyond the specific a priori hypothesis tested here but not completely unexpected. Firstly, there are previous studies that have shown representational patterns of performed finger motor sequences to be distributed across primarily the premotor and posterior parietal cortices, but also to exist in other temporal, occipital, and frontal areas (Kornysheva and Diedrichsen 2014; Yokoi et al. 2018; Pinsard et al. 2019). Secondly, several of the regions illustrated in the present findings, including the inferior parietal lobe, premotor areas, and inferior frontal cortices, are spontaneously engaged also when passively observing or listening to the actions of others, provided that the observer has some knowledge of how to perform the action (Hardwick et al. 2018). Ricciardi and colleagues have demonstrated that this action-observation network develops even in the congenitally blind and can thus learn to represent movements based purely on auditory cues (Ricciardi et al. 2009). The same laboratory has additionally shown, using MVPA, that observed actions are represented supramodally across both the auditory and visual modalities (Ricciardi et al. 2013). Thus, sensory information of different modalities appears to converge on a common abstract form of representation that is used for information processing throughout this network. In line with this, Herholz et al. (2016) demonstrated a causal influence of piano practice on the neural correlates of auditory imagery. As mentioned previously, the notion of a common coding system for perception and action is a core assumption of ideomotor theory (Prinz 1990). The putative neural substrates of this system are the mirror-neurons. “Mirror-neurons” refer to neurons that fire both when an action is performed and when it is merely observed (Rizzolatti et al. 2001). Importantly, mirror-neuron activity during action-observation is dependent on the observer’s previous experience performing the action, which aligns well with ideomotor theory. Key areas of the putative mirror-neuron system in humans are the inferior and superior parietal lobes, superior temporal sulcus, PMD, PMV, and IFG (Rizzolatti and Craighero 2004). All these regions were in the searchlight analysis found to contain informative voxels concerning the ordinal structure of trained auditory(−motor) sequences. Thus, an interpretation of the outcomes with regard to trained melodies is that they were firstly perceived and processed as spatial pitch sequences along the dorsal visual stream (Bengtsson and Ullén 2006) and then, given previous auditory-motor associative learning, transformed to an abstract feature code that was distributed across a network of brain regions involved in planning, simulating, and organizing sequential finger movements (Pinho et al. 2016).

### Effects of Practice and the Role of the Default Mode Network for Transfer

Despite the transfer effect in the experimental group, trained and novel melodies could still be distinguished from one another using MVPA. There was a notable overlap, but also interesting differences between the searchlight maps resulting from classification of trained melodies on the one hand and classification of novel melodies of the other. The most striking difference was that representations of trained melodies were more abundant in the right hemisphere, while representations of novel melodies were found bilaterally and included additional midline regions of the default mode network. A straightforward explanation of these findings is that listening to the trained melodies induced more specific motor-related representations of action, while listening to the novel melodies engaged a wider portion of the action-observation network, as well as default mode regions involved in process and outcome simulations (Gerlach et al. 2014), to solve for the missing sequence-specific information. In support of the first of these two propositions, Wiestler and Diedrichsen (2013) found that sequence-specific motor representations develop and become more distinct with practice. However, since the lateralization here was rightward, the effect of practice was not likely tied to the trained effector. Instead, the observed effect could be viewed in relation to studies suggesting a differential lateralization of spectral/pitch and temporal/rhythmic processing (Patterson et al. 2002; Zatorre et al. 2002; Coffey et al. 2016). Recently, Flinker et al. (2019) proposed that this difference reflects how spectrotemporal modulations are processed—the right and left hemispheres being preferentially sensitive to changes in spectral and temporal features, respectively. As a melody is essentially a series of pitch modulations, it seems natural that processing would tend to become more right-lateralized when for instance piano training tunes perception to melodic content. Further, trained melodies could be classified in the supplementary motor area and putamen, which indicates specificity of processing and that representations included (effector-independent) movement features at the sequence level (Bengtsson et al. 2005; Bednark et al. 2015). The novel melodies were instead more broadly represented across the brain. Interestingly, the searchlight map for novel melodies, which included the IFG, insula, STG, MCC, and default mode regions, matches quite well with the network of regions we have previously found engaged during spontaneous forms of piano improvisation (Pinho et al. 2016). It is conceivable that similar processes used for generating novel melodies in real-time were spontaneously triggered by the novel melodic stimuli in this experiment to simulate action sequences. This introduces an interesting and novel role of default mode regions in motor transfer, which is nonetheless very much in line with their previously described functionality (Gerlach et al. 2014).

## Conclusion

The results in this study collectively provide strong and direct evidence for training-induced sequence-specific action-perception coupling by showing that a series of sensory events, which during skill learning have been perceived as a sequence of action outcomes, can trigger sequence-specific representations in the motor system. The finding that novel melodies could also be classified in multiple brain regions, including default mode regions, is very interesting. As the control experiment confirmed these outcomes to be training-dependent, we suggest that action-perception coupling, by way of the common coding principle, is potentially an important mechanism also for near transfer, as well as for spontaneous action simulation during action observation (Kilteni et al. 2018).

## Supplementary Material

Supplementary_material_bhaa018

## Notes

We thank Prof. Robert Zatorre for valuable discussions and comments on the manuscript, Dr Laura Babcock for programming assistance, and Dr Rita Almeida for consultations on the experimental design.
