# Supplementary material for: Action-Perception Coupling and Near Transfer: Listening to Melodies after Piano Practice Triggers Sequence-Specific Representations in the Auditory-Motor Network
Source: Cereb Cortex. 2020 May 21;30(10):5193–203. doi: 10.1093/cercor/bhaa018 (PMC7472192; doi:10.1093/cercor/bhaa018)

**Table S1. ROI-based classification results in Experiment 1 based on melodic categories (trained versus novel), derived from difference scores of classification accuracy (true scores − chance scores).**

|  |  | LPMD | | |  | RPMD | | |  | LpSTG | | |  | RpSTG | | |
| --- | --- | --- | --- | --- | --- | --- | --- | --- | --- | --- | --- | --- | --- | --- | --- | --- |
|  | r (mm) | Δ (%) | *p* | *d* |  | Δ (%) | *p* | *d* |  | Δ (%) | *p* | *d* |  | Δ (%) | *p* | *d* |
| Trained vs. | 4 | 1.2 | .254 | .20 |  | **5.5** | **.014** | **.98** |  | .6 | .254 | −.21 |  | 2.5 | .054 | .55 |
| Novel | 6 | **3.4** | **.039** | **.68** |  | **4.1** | **.042** | **.64** |  | **2.7** | **.014** | **.95** |  | 2.5 | .072 | .49 |
|  | 8 | **5.4** | **.014** | **1.01** |  | **4.4** | **.035** | **.72** |  | **3.5** | **.014** | **.91** |  | 2.7 | .051 | .48 |

The table displays FDR-corrected *p*-values.

r = ROI radius, Δ = difference score (true − chance accuracy), *d* = effect size (Cohen’s *d*), LPMD = left dorsal premotor area, RPMD = right dorsal premotor area, LpSTG = left posterior superior temporal gyrus, RpSTG = right posterior superior temporal gyrus

**Table S2. ROI-based classification results in Experiment 2 based on melodic categories (trained versus novel), derived from difference scores of classification accuracy (true scores − chance scores).**

|  |  | LPMD | | |  | RPMD | | |  | LpSTG | | |  | RpSTG | | |
| --- | --- | --- | --- | --- | --- | --- | --- | --- | --- | --- | --- | --- | --- | --- | --- | --- |
|  | r (mm) | Δ (%) | *p* | *d* |  | Δ (%) | *p* | *d* |  | Δ (%) | *p* | *d* |  | Δ (%) | *p* | *d* |
| Prelistened vs. | 4 | .8 | .465 | .13 |  | −.4 | .465 | −.08 |  | .2 | .465 | .06 |  | .2 | .465 | .05 |
| Novel | 6 | 3.5 | .165 | .62 |  | 1.3 | .327 | .26 |  | −.1 | .465 | −.03 |  | 1.2 | .310 | .31 |
|  | 8 | 2.3 | .268 | .41 |  | 1.4 | .268 | .37 |  | 1.4 | .165 | .72 |  | 3.3 | .193 | .52 |

The table displays FDR-corrected *p-*values.

r = ROI radius, Δ = difference score (true − chance accuracy), *d* = effect size (Cohen’s *d*), LPMD = left dorsal premotor area, RPMD = right dorsal premotor area, LpSTG = left posterior superior temporal gyrus, RpSTG = right posterior superior temporal gyrus

**Figure S1. Searchlight classification results based on melodic categories (trained versus novel).** Color indicates significant classification accuracy (*z*-scores). R = right hemisphere, L = left hemisphere.


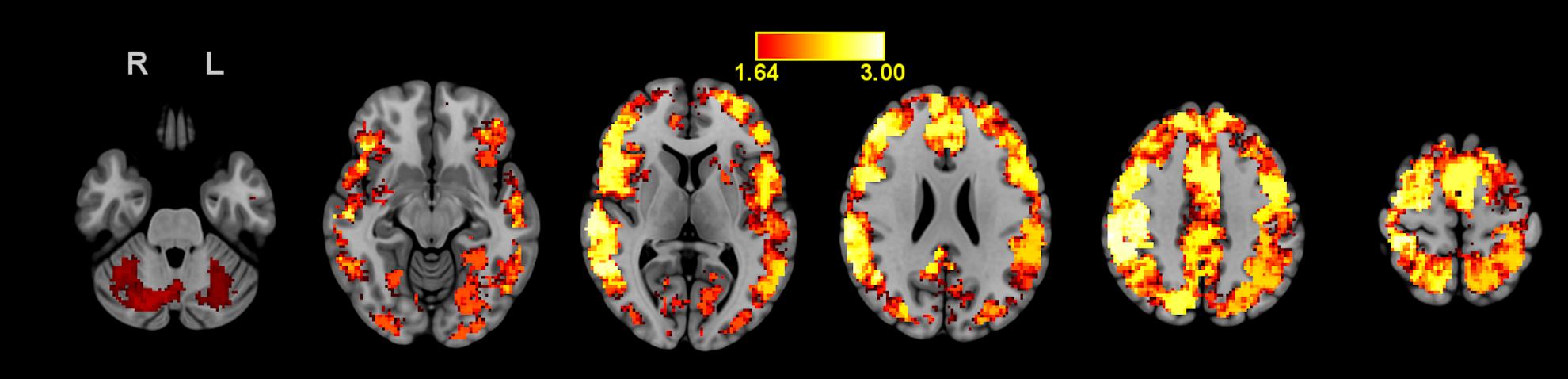

Supplement: Supplementary_material_bhaa018 [file supplementary_material_bhaa018.docx]
